# Early recurrent neonatal tongue glial heterotopia presenting with acute airway obstruction: the first case report

**DOI:** 10.3389/fped.2025.1674740

**Published:** 2026-01-08

**Authors:** Junwei Zhao, Xiangrong Wang, Fan Yin, Jing Chu

**Affiliations:** 1 Department of Stomatology, Anhui Provincial Children's Hospital, Hefei, China; 2 Early Orthodontic Treatment Center, Hefei Stomatological Hospital, Hefei, China; 3 Department of Pathology, Anhui Provincial Children's Hospital, Hefei, China

**Keywords:** complete excision, congenital tongue, glial choristoma, glial heterotopia, tongue glial heterotopia

## Abstract

**Background:**

Glial heterotopia is a rare congenital developmental anomaly characterized by the presence of mature glial tissue outside the central nervous system. It most commonly affects the nasal region, while tongue involvement, particularly at the base of the tongue, is extremely uncommon.

**Case:**

This case represented the first reported instance of early postoperative recurrence of tongue glial heterotopia. We described a 24-day-old male infant diagnosed with tongue glial heterotopia complicated by pneumonia and presenting with airway obstruction. The patient initially underwent conservative local excision of the lesion; however, the lesion rapidly recurred within three weeks postoperatively. A second, more extensive surgical resection with a 5-mm safety margin was subsequently performed. Histopathological examination of both specimens confirmed the diagnosis of glial heterotopia.

**Conclusion:**

This report provided a systematic overview of the diagnostic and therapeutic process, including a one-year postoperative follow-up, during which no recurrence or complications were observed. The clinical course of this case highlighted the potential for early recurrence of tongue glial heterotopia following incompletely conservative excision (with the incision placed along the tumor margin). Our findings suggest that early surgical intervention stabilizes neonates with dyspnea and secures a patent airway. Moreover, extensive resection—defined as extending the incision approximately 5 mm into normal tissue—is essential in tongue glial heterotopia to achieve complete pathological clearance and minimize the risk of recurrence.

## Introduction

Glial heterotopia is a rare, congenital, non-neoplastic condition characterized by ectopic glial tissue located outside the central nervous system. The exact etiology and pathogenesis remain unclear, but proposed mechanisms include abnormal migration or entrapment of glial cells during embryogenesis, displacement of neuroectodermal tissue, herniation and subsequent separation of neural tissue from the brain, remnants of primitive neuroglial elements (1).

Glial heterotopia often presents without obvious symptoms and most commonly affects the nasal region, where it is referred to as nasal glial heterotopia. The estimated incidence of nasal glial heterotopia is approximately 1/20,000 to 1/40,000 live births (2). In contrast, tongue glial heterotopia is extremely rare, with fewer than 30 cases reported globally in the literature (3). Some patients also present with associated congenital anomalies, including congenital heart defects (4). In this report, we present the case of a male neonate with a lesion located at the base of the tongue, which led to upper airway and gastrointestinal obstruction, resulting in suprasternal and supraclavicular retractions, as well as feeding difficulties. Clinical and imaging assessments further revealed neonatal pneumonia, congenital laryngomalacia, and an atrial septal defect, all of which severely affected the infant's growth and development.

To our knowledge, this is the first documented case of early postoperative recurrence of tongue glial heterotopia, with histopathological confirmation of recurrent lesions. We provide a systematic review of the diagnostic and therapeutic course, along with a one-year postoperative follow-up. This case offers valuable insight into the clinical management of this rare entity and highlights the importance of surgical strategy, particularly regarding resection margins, in preventing early recurrence.

## Case report

The child was a full-term male infant born vaginally at another hospital, with a gestational age of 37 weeks + 6 days and a birth weight of 3,100 g. There was no history of resuscitation, and no abnormal tongue mass was noted at birth. From day 2 to day 22 of life, the parents observed inspiratory stridor accompanied by feeding difficulties. The symptoms progressively worsened, but no medical attention was sought. On day 23, the infant presented with severe symptoms and weight loss, prompting an outpatient visit to a nearby hospital. An intraoral examination revealed a mass on the tongue. That hospital subsequently performed an MRI, which reported a posterior mass with normal signal intensity, accompanied by narrowing of the oropharyngeal cavity. On day 24, due to persistent stridor and respiratory distress, the child was transferred to the emergency department of our hospital and subsequently admitted to the neonatology department for further evaluation and treatment. Past medical history: The mother had no anemia during pregnancy and no history of prenatal medication. Family history: The parents denied consanguinity, and there was no known family history of genetic, congenital, or infectious diseases.

**Figure 1:**
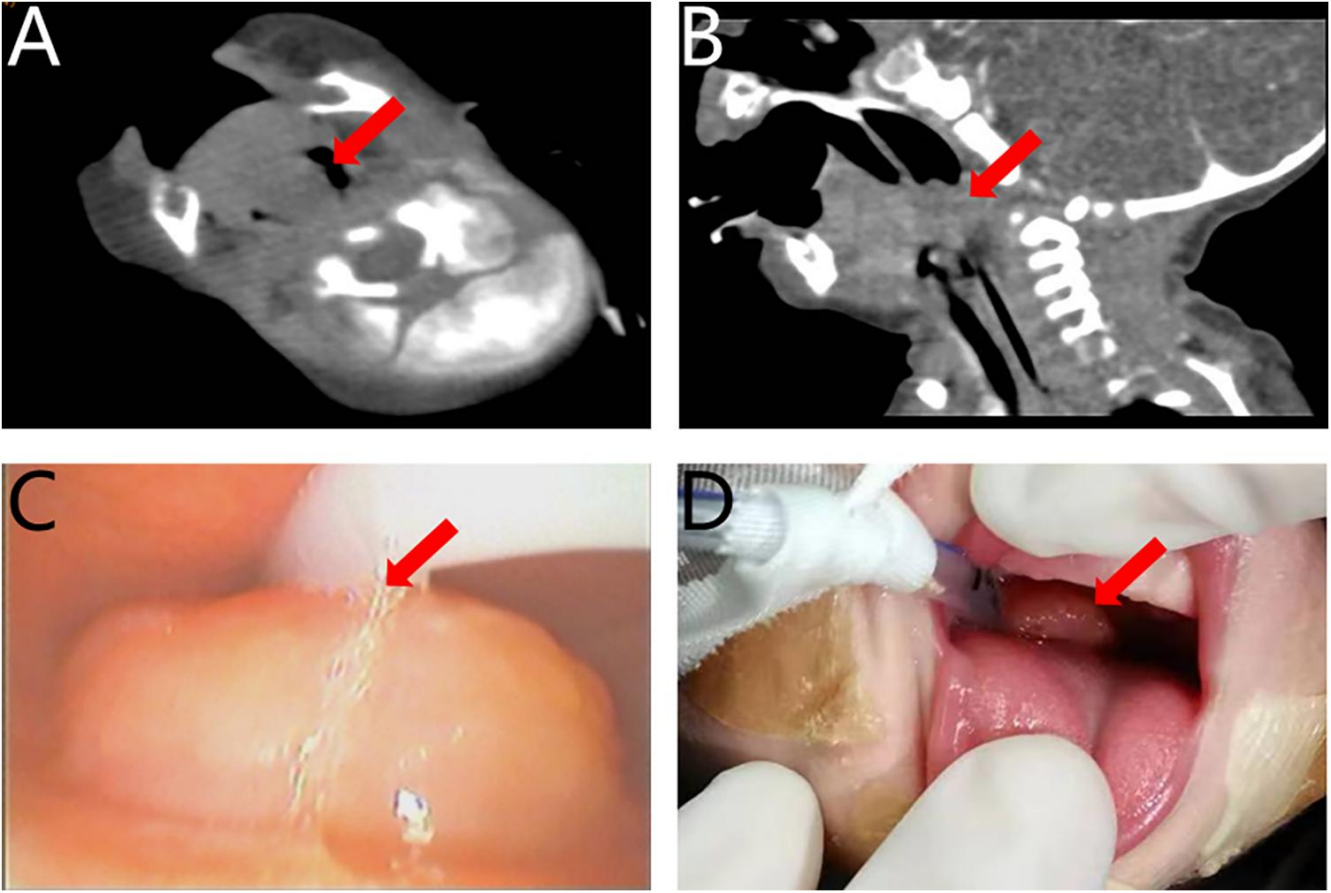
**(A,B)** CT showing the tumor arising from the base of tongue (red arrow). **(C)** Fiberoptic bronchoscopy revealing the tumor at the base of the tongue (red arrow). **(D)** Preoperative intraoral view showing a mass at the tongue base (red arrow).

Comprehensive physical examination revealed no apparent abnormalities or associated congenital anomalies. And his weight upon admission was 2,600 g. Intraoral examination revealed no teeth, no cleft palate, and a spherical mass measuring approximately 2.0 cm × 2.0 cm located posterior to the tongue base. The mass was pink, smooth-surfaced, firm in consistency, non-compressible, non-pulsatile, and had a broad base attached to the tongue root, obstructing the oropharyngeal airway. Auxiliary examination: the child had already undergone MRI at the previous hospital, so we did not repeat the examination. Unfortunately, the parents only brought the MRI report to the consultation, and we were unable to obtain the images and detailed information. Contrast-enhanced CT revealed a plaque-like, slightly hypodense lesion at the base of the tongue, with well-defined borders and no evident communication with adjacent structures (Figures 1A,B). The center of the lesion showed low density without significant enhancement, and the airway was notably compressed. Fiberoptic bronchoscopy revealed a dense mass at the base of the tongue, completely obstructing the post-palatal airway space (Figure 1C). The infant was also diagnosed with congenital laryngomalacia (Olney Type III), characterized by posterior displacement of the epiglottic base. Ultrasound examination of the submandibular and cervical regions revealed an anechoic mass in the floor of the mouth and tongue base, with well-defined borders, regular shape, and no internal septation. The thyroid gland appeared normal in size and shape, with homogeneous echotexture. Chest CT revealed increased bronchovascular markings and patchy high-density shadows in both lungs, consistent with neonatal pneumonia. Neonatal blood work, including CBC, coagulation, and biochemical tests, infectious disease tests, showed no abnormalities. Following consultation with otolaryngology, the neonatologist rendered a provisional diagnosis: 1. cyst at the tongue? (The nature of the lesion remains to be confirmed by histopathology. Differential diagnoses included thyroglossal cyst, dermoid cyst, hemangioma, chondroid, osseous, or glial heterotopia, ectopic thyroid of the tongue, and benign teratoma, whereas malignant tumors such as rhabdomyosarcoma or fibrosarcoma were considered less likely.); 2. congenital laryngomalacia (Olney Type III); 3. neonatal pneumonia.

On hospital day 1, the infant was admitted to an incubator, received nasogastric feeding and supplemental oxygen to maintain adequate oxygen saturation, and was started on antimicrobial therapy for pneumonia. On hospital day 3, the infant developed progressive dyspnea accompanied by suprasternal, intercostal, and subcostal retractions (three-concave sign), requiring emergency tracheal intubation and mechanical ventilation. The mass at the base of the tongue was visible intraorally during the intubation procedure (Figure 1D). On hospital day 7, after one week of antimicrobial therapy, the infant remained clinically stable. To minimize repeated exposure to general anesthesia and to effectively relieve persistent airway obstruction, the otolaryngology and center of endoscopy teams planned a combined procedure: holmium-laser tumor excision under fiberoptic bronchoscopy, followed by concurrent supraglottoplasty to address congenital laryngomalacia.

**Figure 2:**
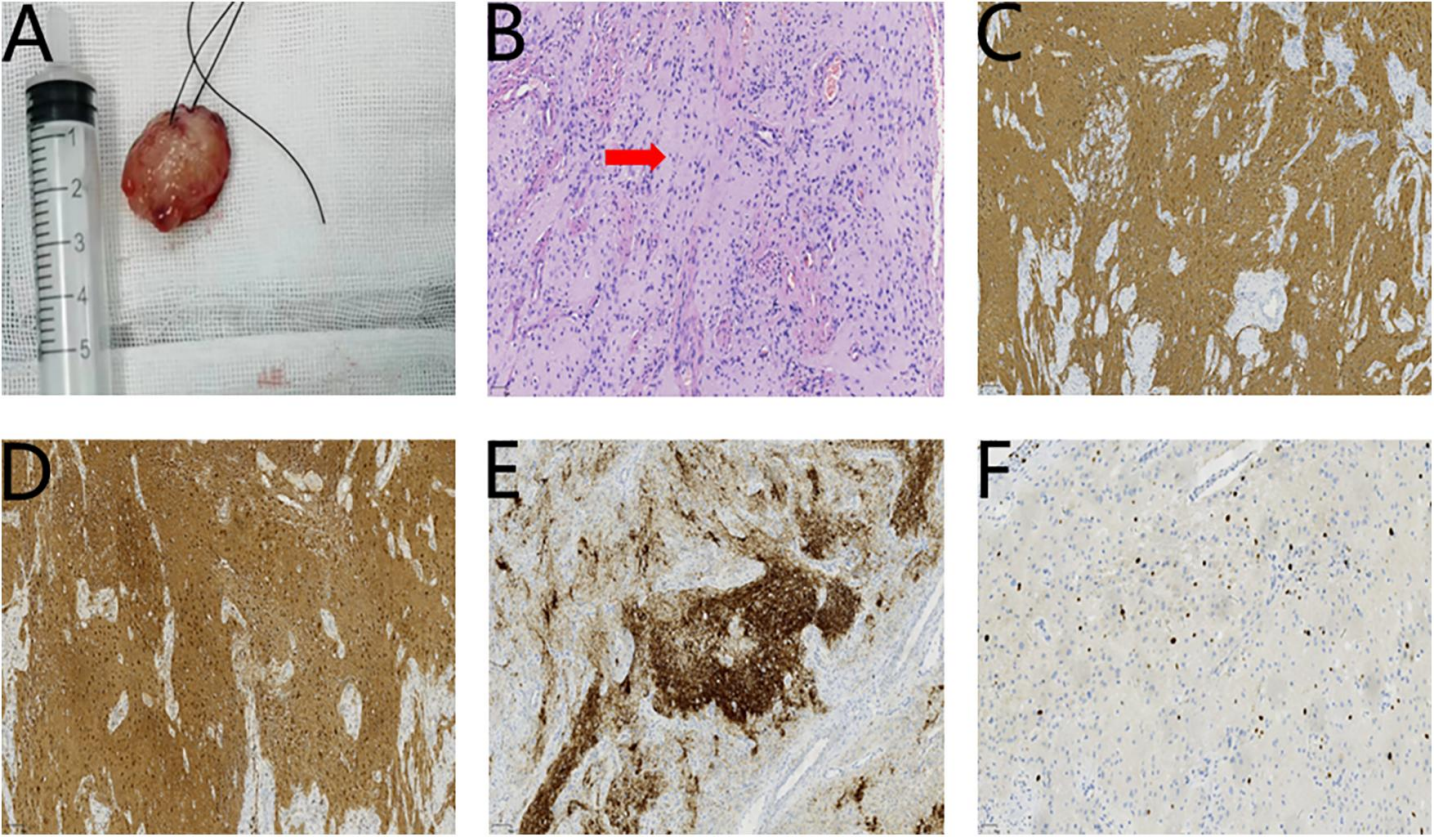
**(A)** The first surgical specimen. **(B)** The first HE staining Original magnification (red arrow) (×200). The findings of the first immunohistochemistry: **(C)** GFAP(+++) (×100). **(D)** S100(+++) (×100). **(E)** Syn(++) (×100). **(F)** Ki-67 (15%) (×200).

Under general anesthesia, the bronchoscope encountered substantial resistance while passing through the nasal cavity into the posterior nasal aperture and pharyngeal space, resulting in limited visualization of the mass. The otolaryngology and endoscopy teams subsequently adopted an oral approach. Due to the tumor's large size, firm consistency, and technical difficulties, the use of a holmium laser was abandoned. Considering the tongue's complex anatomy and surgical safety, a multidisciplinary team determined that the oral and maxillofacial surgery team would perform the resection using a scalpel assisted by electrocautery. Initially, the tongue was externally retracted with a suture to expose the mass, and an additional suture was placed through the lesion for stabilization. Subsequently, given the large tumor volume, a spindle-shaped incision confined to the tumor margin was designed, followed by circumferential infiltration of 2% lidocaine with 1:200,000 epinephrine to minimize bleeding. Next, the firm, well-demarcated mass was excised starting with a scalpel along the margin and completed with an electrosurgical knife, while preserving lingual vessels and achieving hemostasis with electrocoagulation (Figure 2A). Then, the incision was closed using 5-0 absorbable interrupted sutures. Post-resection, the posterior nasal cavity and pharyngeal space were patent. The otolaryngology and endoscopy teams utilized transnasal bronchoscopy with multi-site holmium laser ablation, which elevated the epiglottis and relieved the obstruction. Postoperatively, the child was returned to the ward without endotracheal intubation. He exhibited mild tachypnea and stridor, with a transcutaneous oxygen saturation of 90%. Symptomatic supportive care was provided, including nasal cannula oxygen and intravenous methylprednisolone to reduce airway edema. By postoperative day 2, spontaneous breathing had largely normalized, with oxygen saturation exceeding 95%, while supplemental oxygen via the incubator was maintained.

**Figure 3:**
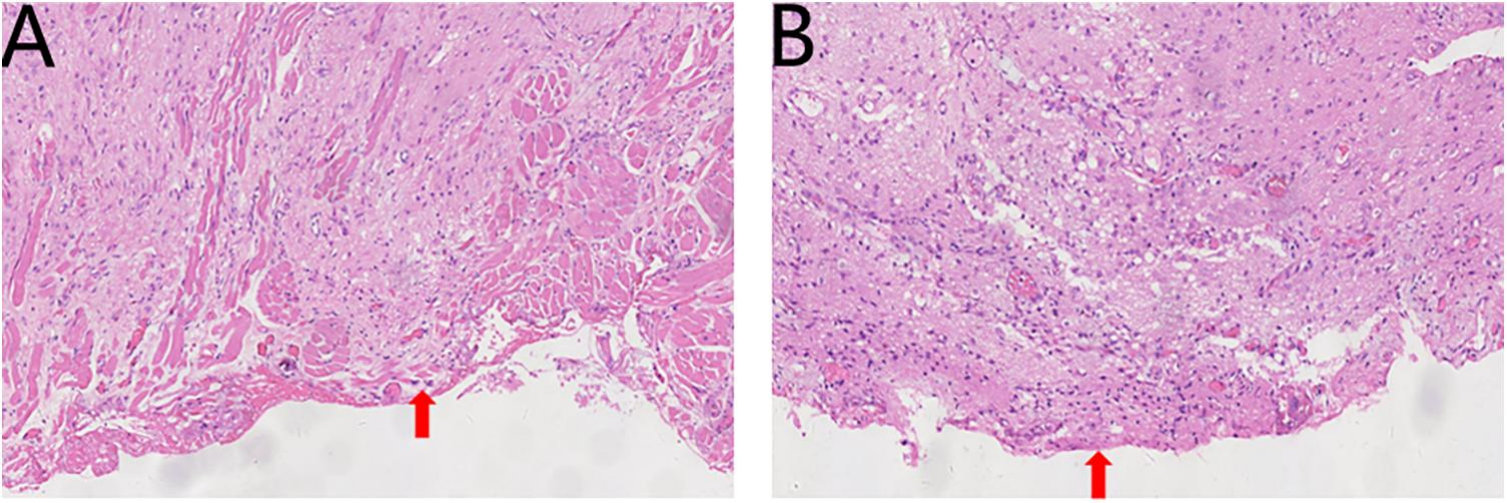
**(A,B)** HE staining of the first surgical specimen (×100) showing residual glial heterotopia at the margins (red arrow).

Histopathological analysis of the excised specimen (measuring 2.2 cm × 2.0 cm × 1.0 cm) revealed sparsely arranged glial cells, and inflammatory granulation tissue hyperplasia, consistent with glial tissue morphology (Figure 2B). Immunohistochemistry findings (Figures 2C–F): glial fibrillary acidic protein (GFAP) (+++), S100(+++), synaptophysin (Syn) (++), Ki-67 (15%). Definitive diagnosis: tongue glial heterotopia. Unfortunately, hematoxylin and eosin (HE) staining of the incision margin revealed residual glial heterotopia tissue; therefore, the possibility of a second operation could not be excluded (Figures 3A,B).

**Figure 4:**
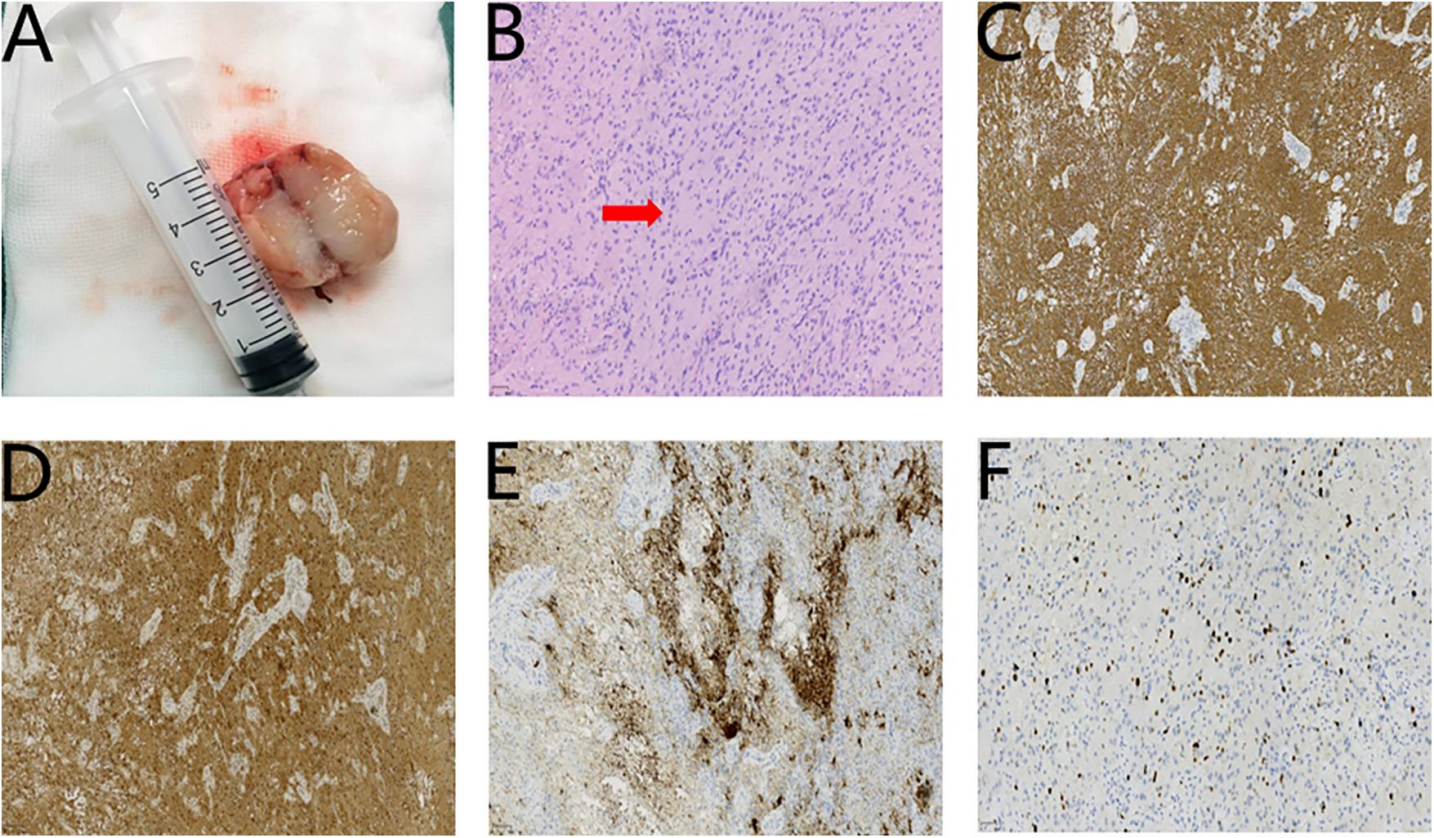
**(A)** The second surgical specimen. **(B)** The second HE staining Original magnification (red arrow) (×200). the findings of the second immunohistochemistry: **(C)** GFAP(+++) (×100). **(D)** S100(+++) (×100). **(E)** Syn(++) (×100). **(F)** Ki-67 (17%) (×200).

**Figure 5:**
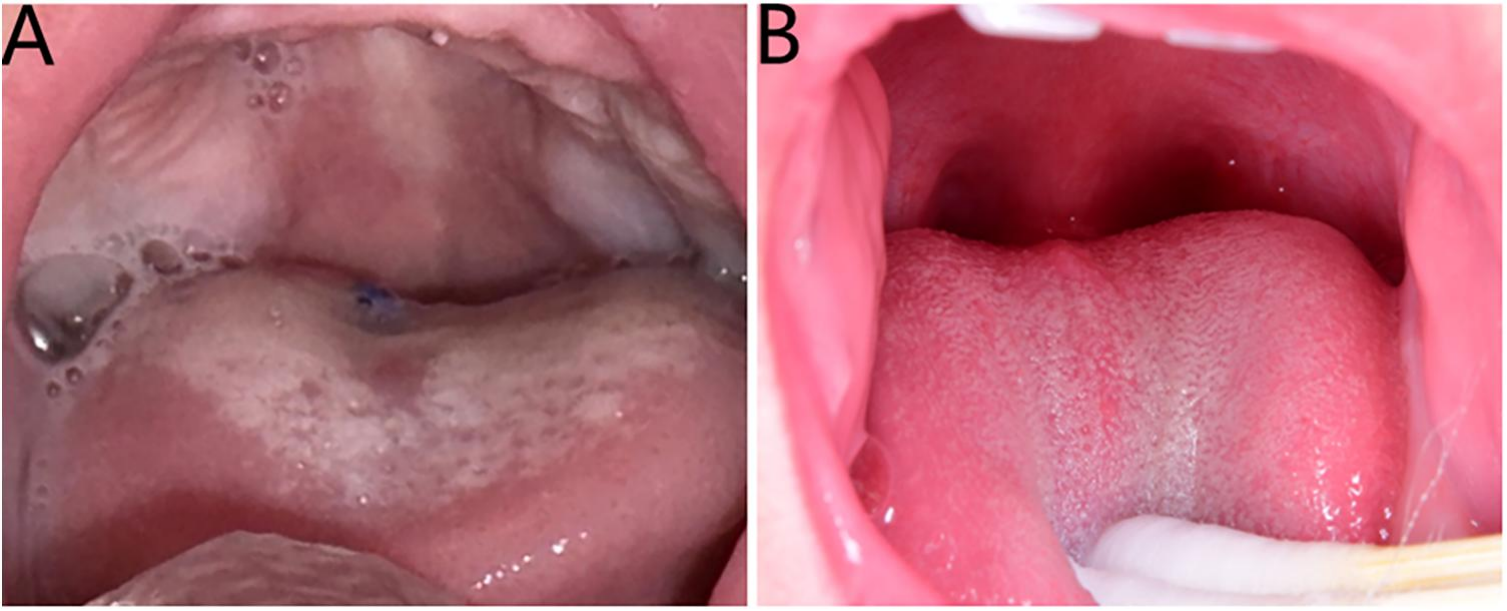
**(A)** No recurrence at 1-month follow-up. **(B)** No recurrence at 1-year follow-up.

Follow-up bronchoscopies at 1 and 2 weeks postoperatively showed a small amount of grayish-white pseudomembrane on the tongue surface. The epiglottis elevated smoothly, with normal vocal cord and posterior glottic function. However, by the third postoperative week, the glial heterotopia recurred at the original location, causing developed dyspnea accompanied by the three-concave sign again. An emergency second surgery was performed by the oral surgery team. The second operation followed the same procedure as the initial surgery; however, to prevent recurrence from incomplete resection, the incision was extended 5 mm into normal tissue surrounding the tumor and carried down to the tongue muscle layer to achieve an expanded excision of the glial heterotopia (Figure 4A). Postoperatively, the infant was transferred to the ward with the tracheal tube in place and received ventilator-assisted ventilation for one day, maintaining oxygen saturation above 95%. The resected mass measured 3.2 cm × 2.4 cm × 1.6 cm. Histopathology and immunohistochemistry results were consistent with the first specimen (Figures 4B–F): GFAP (+++), S100 (+++), Syn (++), Ki-67(17%). The second Final diagnosis is same as the first and no residual glial heterotopia tissue at the edge of the incision. The patient was followed up in-hospital for two weeks, during which the tongue wound healed well and airway patency was restored. At the one-year postoperative follow-up, no recurrence was observed, and the tongue had completely healed without any complications (Figures 5A,B). At this time, the child's height and weight were within the normal range for age. Although the child's language development was still in progress, he could pronounce simple words like “mama” and “dada”, indicating no abnormalities in speech or phonation. According to the parents' report, the child's daily swallowing function was normal.

## Discussion

Glial heterotopia may present as either a solid or cystic mass and is extremely rare when located at the base of the tongue in neonates (5). Clinical and radiologic differentiation from other congenital lesions—such as ectopic thyroid tissue, tongue thyroglossal duct cysts, or teratomas—can be particularly challenging. Although imaging features of glial heterotopia are nonspecific, radiologic evaluation plays a critical role in determining the lesion's anatomical location, extent, and relationship to adjacent structures, as well as in surgical planning and risk assessment. These lesions commonly arise along extracranial midline locations and typically exhibit homogenous signal intensity with relatively well-defined margins on imaging studies. Unfortunately, the parents only provided the MRI report during consultation, so the actual images and detailed information were unavailable. Nevertheless, the findings from contrast-enhanced CT and fiberoptic bronchoscopy largely supported our initial diagnosis for this infant. Due to the rarity of this condition, a definitive diagnosis is often not possible preoperatively. In the present case, the final diagnosis of glial heterotopia was established by histopathological examination following surgical excision.

A review of previous literature indicates that the postoperative recurrence rate of nasal glial heterotopia ranges from 4% to 10% (6, 7). Regarding glial heterotopia of the tongue, a 1922 German case mentioned postoperative recurrence (8); however, the original article is inaccessible to us. García-Prats (9) summarized some information from that report, including the patient's sex, age, and lesion size, but without detailed data. Consistent with Kirikoshi's viewpoint (3), we remain skeptical about the validity of the recurrence case described in that article. The case reported by Ofodile (10) involved surgical excision of a large lesion in a female infant at 4 days old. Clinical examination revealed a 6 × 5 cm grayish mass occupying most of the anterior two-thirds of the tongue dorsum. Intraoperatively, a small (2 mm) adjacent lesion approximately 1 cm anterior to the main mass was noted but left untreated. This adjacent lesion enlarged postoperatively and was resected at 1 month of age; follow-up at 10 months showed no recurrence.

We do not consider this a true recurrence of glial heterotopia because the enlargement occurred in adjacent tissue rather than at the original lesion site, and the adjacent lesion was not pathologically confirmed as glial heterotopia. Therefore, our viewpoint aligns with that of Kirikoshi (3), as there have been no previous reports of recurrence in cases of glial heterotopia of the tongue prior to this case. Histopathology remains the gold standard for diagnosing glial heterotopia. In this report, recurrence of the glial heterotopia at the tongue was confirmed histopathologically, with the recurrent lesion located at the original site and exceeding the size of the initial lesion. This finding holds significant implications for the future surgical management of this rare condition.

Regarding the cause of recurrence in this patient, we discuss two main aspects. Firstly, current histological and pathological studies indicate that glial heterotopia can exhibit rapid growth (11). Most reported cases of ectopic brain tissue are primarily composed of glial tissue (12, 13). Astrocytes dominate in most cases and often constitute the sole cellular component (14, 15). Other mixed morphologies may include ependymal-lined clefts, choroid plexus, and retinal structures (13, 16). Once the ectopic glial tissue differentiates towards neural derivatives, the lesion cell population may proliferate synchronously with the normally located brain tissue, which grows most rapidly during the first two years after birth (17, 18). This may explain the rapid growth and potential recurrence of glial heterotopia in the tongue during the neonatal period. Furthermore, the Ki-67 indices of 15% and 17% in the two pathological examinations in this report also indicate a proliferative potential of the lesion. In addition, within the central nervous system, astrocytes respond to significant tissue injury by reactive astrocytosis, where damaged cells re-enter the cell cycle, leading to tissue proliferation (19–21). From a pathological perspective, we speculate that in neonatal patients, surgical resection might act as a pathological stimulus for the rapidly growing glial heterotopia if the lesion is not completely and thoroughly removed. Secondly, we hypothesize that the scope of surgical resection is an important factor influencing recurrence. In this report, the two surgeries involved different resection margins. Considering the patient was a neonate and the glial heterotopia occupied a large portion of the tongue, the initial surgery prioritized avoiding damage to critical surrounding structures and therefore did not involve an extended resection. During the first surgery, a conservative complete excision was performed; however, HE staining of the incision margin revealed residual glial tissue, suggesting that proliferative remnants remained. During the second surgery, the excision margin was extended by 5 mm to ensure complete removal of the lesion. HE staining of the incision margin confirmed the natural tissue, and no recurrence was observed during one year of follow-up. This case highlights that, in the management of tongue glial heterotopia, extensive resection at the initial surgery is crucial, with postoperative pathological confirmation of tumor-free margins. Incomplete excision is likely to result in early recurrence. Currently, there is no consensus on the resection margin for tongue glial heterotopia. In neonates, most reports advocate for an extended complete excision ranging from 2 mm to 1 cm (9, 22, 24), but a report describe partial excision of lesions, although not in neonates (3). Based on this case, we recommend extended resection for neonatal tongue glial heterotopiadue to the indistinct boundary between lesion and normal tissue, as conservative excision may lead to recurrence. The extent of surgical margin extension should be tailored to the tumor size. For larger lesions, a relatively narrower margin may be appropriate. Based on current evidence, a 5-mm margin appears to be optimal. The timing of surgical intervention for glial heterotopia remains controversial. The standard treatment in most cases is early and complete excision of the lesion. However, for lesions not affecting the patient's breathing or feeding, some clinicians support delayed surgery to minimize operative damage and complications (23).

Furthermore, the neonate presented with the rare coexistence of glial heterotopia and congenital laryngomalacia. After comprehensive assessment of airway obstruction etiology, anesthetic risk, and surgical burden, the teams elected to perform supraglottoplasty concurrently with tumor resection, despite the increased surgical complexity. Preoperatively, these teams planned to use the holmium laser as the primary resection tool, enabling dual procedures with a single device while maintaining high safety. However, the large lesion volume and firm tissue consistency, distinct from typical cysts, limited the efficacy of holmium laser excision. Following multidisciplinary discussion, oral and maxillofacial surgeons proceeded with conventional tumor resection assisted by an electrosurgical unit, and the same approach was used for the second operation. Electrocautery, which achieves tissue cutting via thermal effects, has become widely adopted in oral surgery due to its low technical sensitivity and rapid tissue removal (24). However, it is limited by reduced precision and considerable thermal damage to adjacent tissues. In contrast, laser therapy vaporizes tissues for cutting, allowing precise, minimally invasive procedures with minimal collateral injury (25), though it is less efficient for larger tissue resections. Currently, reported cases of tongue gliotic heterotopia are predominantly treated using electrocautery-assisted techniques (4, 11). Case analysis suggests that large, solid tongue gliotic lesions benefit from electrocautery-assisted resection to achieve faster removal and minimize tissue trauma when externally exposed, whereas smaller tumors may be more appropriately managed with laser therapy.

## Conclusion

We reported for the first time a case of early postoperative recurrence of glial heterotopia located at the base of the tongue, a phenomenon not previously documented. This case report provided valuable reference for the future surgical management of this rare condition. Based on this case and a review of the literature, we recommend that Resolving pneumonia prior to the definitive procedure is essential to provide an optimal surgical environment, and early surgical intervention stabilizes neonates with dyspnea and secures a patent airway. Moreover, extensive resection—defined as extending the incision approximately 5 mm into normal tissue—is essential in tongue glial heterotopia to achieve complete pathological clearance and minimize the risk of recurrence.

## Data Availability

The original contributions presented in the study are included in the article/Supplementary Material, further inquiries can be directed to the corresponding author.
